# Reconfigurable Magneto‐Optoelectronic Devices for Multidimensional Optical Neural Network

**DOI:** 10.1002/smsc.202500487

**Published:** 2025-12-07

**Authors:** Haiyan He, Yuan Cheng, Wenxuan Zhu, Jiacheng Sun, Jiaming Sun, Tonglu Wang, Cheng Song, Feng Pan, Junying Zhang, Yuyan Wang

**Affiliations:** ^1^ Beijing National Research Center for Information Science and Technology School of Integrated Circuits Tsinghua University Beijing 100084 China; ^2^ School of Physics Beihang University Beijing 100191 China; ^3^ School of Artificial Intelligence Shanghai Jiao Tong University Shanghai 200030 China; ^4^ Key Laboratory of Advanced Materials (MOE) School of Materials Science and Engineering Tsinghua University Beijing 100084 China

**Keywords:** 2D magnetic materials, FeCl_2_, magneto‐optoelectronics, optical neural network, photogalvanic effect

## Abstract

Optical neural networks (ONNs) with extremely low latency, low power consumption, and high parallelism, provide an advantageous computational paradigm to address the rapid development of artificial intelligence. Conventional ONNs focus on handling limited information dimensions such as optical amplitude and phase, which are confined to simple small‐size image classification, raising demand for significant reconfigurability on the perception of inherent high‐dimensional light information. Herein, magneto‐optoelectronic devices with polarization sensitivity are theoretically proposed to construct the ONN with high‐performance multidimensional recognition, which is composed of 2D magnetic half‐metal FeCl_2_ and 2H‐WSe_2_. Polarization sensitivity with photogalvanic effect originates from the space‐inversion symmetrical breaking of 2H‐WSe_2_, yielding the multidimensional perception under zero power consumption. The switchable magnetic configuration of two FeCl_2_ contacts with unique half‐metal band structures nonvolatilely modulates the amplitude and polarity of photoresponse across the wavelength from ultraviolet to near‐infrared. By leveraging multidimensional light encoding, the proposed ONN architecture conducts negative value and nonlinear computations in polarization domain through highly reconfigurable magneto‐optoelectronic mechanisms, which achieves up to 93.5% accuracy across complex tasks including 3D object classification, time‐series recognition, etc. This work illuminates the potential of magneto‐electronics, which extends the applications of ONNs in the real world.

## Graphical abstract

A reconfigurable magneto‐optoelectronic device with polarization sensitivity is proposed to construct multidimensional optical neural networks (ONNs). By combining 2H‐WSe_2_ and 2D half‐metal FeCl_2_ with ideal spin filtering effects, perception of multidimensional optical information is achieved under zero power consumption, which is significantly manipulated by magnetic configurations. Implemented ONNs demonstrate exceptional capacity in solving complex vision tasks, including 3D object classification.

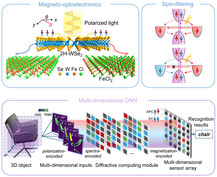

## 1 Introduction

Based on light as the carrier for the transmission and processing of information, optical neural networks (ONNs) benefit from low latency, low power consumption, high throughput, and high parallelism during the processing of light signals.^[^ ^1^, ^2^, ^3^ ^]^ Compared to their electronic counterparts,^[^ ^4^, ^5^, ^6^ ^]^ ONNs have offered a transformative paradigm for artificial intelligence (AI) applications, enabling high energy efficiency and ultrafast computational speed, particularly in face of the continuous and explosive increase of data volume. The high compatibility of ONNs with the on‐chip integration is also demonstrated.^[^ ^7^, ^8^, ^9^ ^]^ Although light carries massive information across multiple dimensions, including intensity, wavelength, polarization, and phase, ONNs are primarily used for the recognition of simple small‐size images in traditional machine vision systems,^[^ ^10^, ^11^ ^]^ falling short in processing the advanced multidimensional tasks in the real world. Additionally, challenges remain in achieving nonlinearity and reconfigurability, which diminishes their capacity in processing complex computations.^[^ ^12^, ^13^ ^]^ Such inadequacy stems from the capabilities of photodetectors, which can be addressed by the perception of inherently high‐dimensional light signals.^[^ ^14^, ^15^ ^]^ However, strategies for achieving significant reconfigurability in photodetectors with the sensitivity of multidimensional optical information remain elusive, thereby hindering the application of ONNs in complex real‐life scenes.

The interplay between magnetic and optical properties offers an advantageous avenue for controlling the response to light signals.^[^ ^16^, ^17^, ^18^ ^]^ Especially, leveraging the widely recognized high stability and high speed in the control of magnetization leads to the development of magneto‐optoelectronic devices.^[^ ^19^, ^20^, ^21^, ^22^ ^]^ Based on the mechanisms including magnon‐exciton coupling,^[^ ^17^, ^23^ ^]^ spin filtering,^[^ ^19^, ^20^, ^22^ ^]^ and magneto‐band structure effects,^[^ ^21^ ^]^ both the exciton spectra and the photoresponse can be determined by the magnetic configurations, serving as the basis of constructing reconfigurable photodetectors. The fundamentals of these mechanisms typically reside in the spin‐dependent energy bands, implying that the reconfigurability is intimately connected to the spin polarization rate. In 2D magnetic half‐metals,^[^ ^24^ ^]^ the spin‐majority bands intersect the Fermi level with a bandgap formed between spin‐minority bands, creating 100% spin polarization rate.^[^ ^25^, ^26^ ^]^ Consequently, the strongest interplay between magnetic and optical properties is anticipated through the combination of 2D magnetic half‐metals and 2D semiconductors, with promising significant reconfigurability. Meanwhile, the absence of dangling bonds on 2D surfaces will guarantee such strong interplay across the atomically fine interface in all‐2D heterostructures.^[^ ^27^, ^28^, ^29^ ^]^

In this work, to achieve high‐performance multidimensional recognition by ONNs, we theoretically propose a highly reconfigurable magneto‐optoelectronic device sensitive to high‐dimensional optical signals including intensity, wavelength, and polarization. Such design of magneto‐electronic device can be accomplished by the combination of 2D semiconductor with space‐inversion symmetrical breaking (monolayer 2H‐WSe_2_)^[^ ^30^ ^]^ and 2D ferromagnetic half‐metal (FeCl_2_),^[^ ^31^ ^]^ which contribute to the high‐dimensional perception and reconfigurability, respectively. Attributed by the spin filtering effect and the unique band structures of FeCl_2_, both the amplitude and polarity of the polarization‐dependent photoresponse can be nonvolatilely modulated by the magnetic configuration in the wavelength ranging from ultraviolet to the near‐infrared. The photogalvanic effect (PGE) stemming from the broken symmetry also enables the operation without bias voltage, bringing about zero power consumption.^[^ ^32^ ^]^ ONN based on our magneto‐optoelectronics achieves high performance in the recognition of 3D objects, promoting a higher accuracy of 93.5% compared with vanilla ONNs. Our work highlights the promising potential of magneto‐optoelectronics, paving the way for enhanced applications of ONNs in real world scenarios.

## 2 Results and Discussion

### 2.1 Design of Magneto‐Optoelectronics for High‐Dimensional ONNs

**Figure 1:**
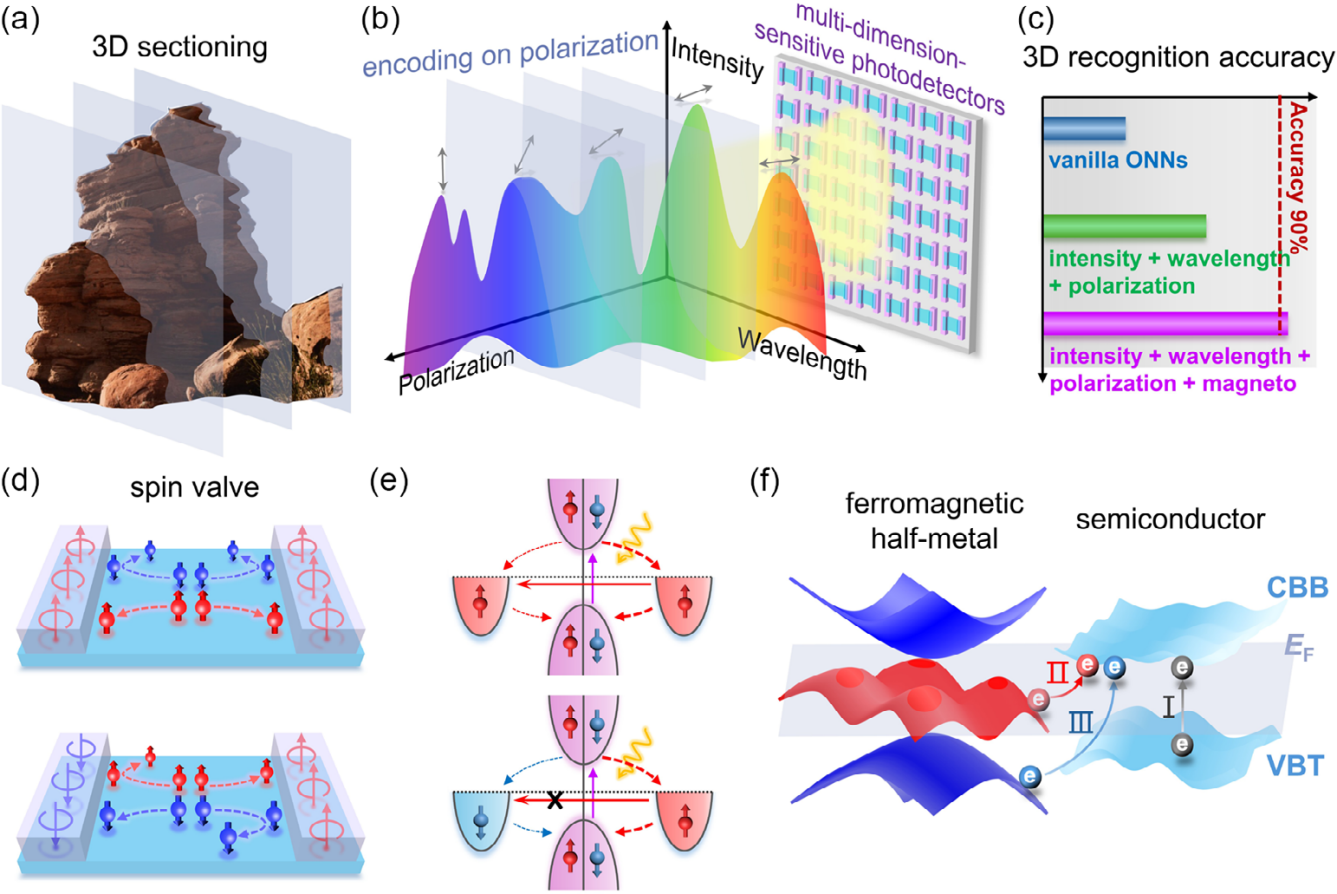
Design of magneto‐optoelectronics with high‐dimensional sensitivity for 3D recognition. a,b) Schematics of the processing of 3D information based on multidimension‐sensitive photodetectors. c) Comparison of the capacity among vanilla ONNs, ONNs with multidimensional sensitivity, and ONNs with both multidimensional sensitivity and magneto‐photoresponse on the recognition of 3D objects. d) Spin filtering in spin valve. The blue and red arrows represent opposite magnetizations. The blue and red balls represent carriers with opposite spin polarizations. e) Schematic carrier transfer in the spin valve with PGE and light irradiation. The parallel and antiparallel spin configurations are shown in the top and bottom panel, respectively. f) Transition modes in the heterostructure composed of ferromagnetic half‐metal and semiconductor. Spin‐degeneracy bands of the semiconductor, including the CBB and the VBT, are rendered in light blue. Spin‐majority and spin‐minority bands of the half‐metal are rendered in red and blue, respectively. The Fermi level (*E*_F_) is represented by the gray plane. The unpolarized, spin‐up and spin‐down electrons are shown by gray, red, and blue balls, respectively.

As schematically shown in **Figure** **1**a, with the perception of high‐dimensional light information, the 3D information of objects can be sectioned into multiple 2D information and encoded on the feature quantity of polarization of optical signals. With the multidimensional sensitive photodetectors performing as the optical sensing layer in the ONN (Figure 1b), such encoded information can be perceived simultaneously. Consequently, the multidimensional recognition ability will be largely enhanced compared to the operation by vanilla ONNs (Figure 1c). Furthermore, by combining two 2D ferromagnetic half‐metals with 2D nonmagnetic semiconductors, a lateral spin valve with photosensitivity is constructed, where the transport of spin‐dependent carrier can be nonvolatilely controlled by the magnetic configuration of two ferromagnets.

As illustrated by Figure 1d, the spin filtering effect enables the carriers with the same spin polarity as the ferromagnet to be transported and those with the opposite spin polarity to be reflected. When the two magnetizations are parallel to each other, forming parallel configuration (PC), the carriers with the same spin polarity are transported towards two ferromagnets. The situation is reversed when the magnetizations are antiparallel to each other, forming antiparallel configuration (APC), bringing about the opposite spin‐polarized carriers at two leads. When the semiconductor possesses PGE, the unpolarized electrons from the valance bands of semiconductor channel are excited to the conductance bands with light stimulus. The broken symmetry enables the different probability of electrons flowing toward two ferromagnets, resulting in spin‐polarized photocurrent at zero bias.^[^ ^33^ ^]^ At the same time, spin‐polarized electrons from the ferromagnet will fill the left holes in the valance bands (Figure 1e). In PC, the spin‐polarized current flowing between two ferromagnets is allowed by the same spin polarization of electrons at the two leads. The situation is opposite in APC due to the opposite magnetization of the two ferromagnets. The spin‐polarized carrier transported from one ferromagnet to the other is restricted, achieving the photoresponse modulated by the magnetic configurations, which provides the bipolar weights for neural networks. The 100% spin polarization rate in half‐metals will maximum the spin filtering effect and the resultant reconfigurability.

The unique band structures of half‐metals, whose Fermi level is fully occupied by the spin‐majority, tightly connects the hopping of different types of carriers with the spin polarization. Correspondingly, the modulation of photoresponse will be enriched through the band alignment in the heterostructure. Figure 1f shows the schematics of representative three transition modes by taking electrons as an example. Primarily, the most fundamental mode is the hopping of unpolarized electrons from the valance bands of the semiconductor to the conductance bands by the excitation of light (mode I), which is the general origin of photoresponse. Further, in the heterostructure, the transition of fully polarized electrons from the half‐metal to the semiconductor possesses a distinct energy gap related to spin polarization. The hopping of spin‐up electrons to the conduction bands of semiconductor shows a smaller energy gap (mode II) compared with the intrinsic band gap of semiconductor, extending the perception to longer wavelength. In contrast, larger photon energy is required for the hopping from the spin‐down bands of half‐metal to the conduction bands of semiconductor (mode III). Such differences will bring about the variation of magneto‐photoresponse with the changing of light wavelength. Equipped with abundant reconfigurability, the capacity of ONN will be further prompted by the magneto‐optoelectronics (Figure 1c).

### 2.2 Magneto‐Photoresponse with Polarization Sensitivity

**Figure 2:**
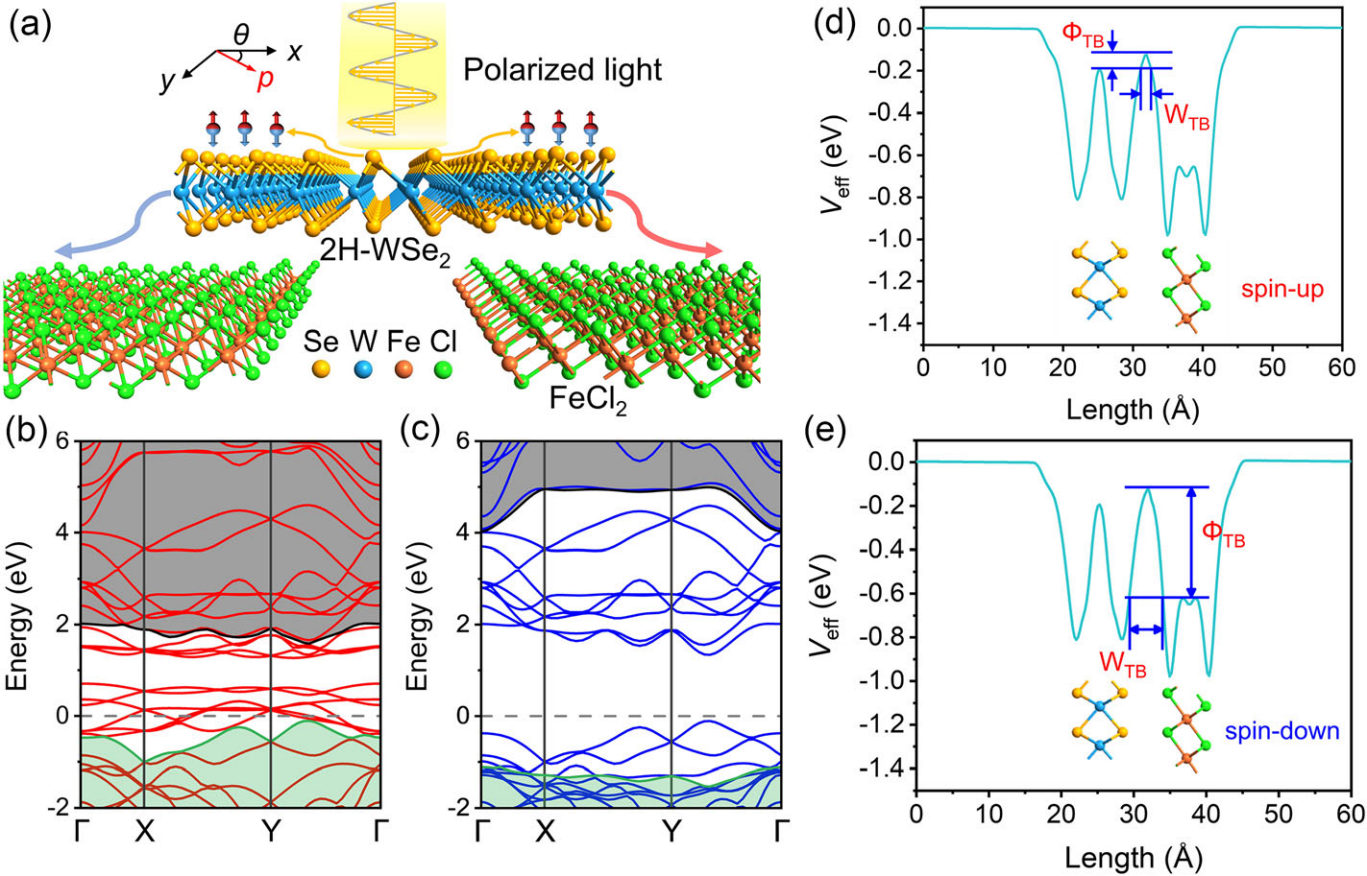
Spin filtering effect in WSe_2_/FeCl_2_ heterostructure. a) Atomic structure and operation principle of magneto‐optoelectronic devices with polarization‐ sensitivity composed of FeCl_2_ as the contacts and 2H‐WSe_2_ as the channel. The generated photocurrent is along *x* direction. The polarization angle of linearly polarized light is defined by *θ*. The spin‐up and spin‐down photocurrent is shown in red and blue, respectively. b,c) Band structures of spin‐majority (spin‐up) and spin‐minority (spin‐down) in WSe_2_/FeCl_2_ heterostucture. The Fermi level is set as zero, shown by the dashed line. The CBB and VBT used to define the Schottky barrier are highlighted by black and green, respectively. d,e) Effective potential of spin‐up and spin‐down electrons in WSe_2_/FeCl_2_ heterostructure. The atomic structure is shown in the inset.

According to the above design, 2H‐WSe_2_ is chosen as the semiconductor layer with 1T‐phase FeCl_2_ as the half‐metal layer. The top and side view of FeCl_2_/WSe_2_/FeCl_2_ device is shown in Figure S1, Supporting Information. As exhibited by the atomic structures in **Figure** **2**a, the mirror symmetry of *y*‐*z* plane is broken in 2H‐WSe_2_. The reduced space‐inversion symmetry brings about the dependence of optical absorption on the polarization of linearly polarized light, contributing to the polarization sensitive photoresponse. The low symmetry can induce PGE, enabling the generation of photocurrent without bias voltage. By combining 2H‐WSe_2_ with two FeCl_2_ layers as the contact electrodes in Figure 2a, the photodetector with the perception of polarization and zero power consumption is constructed, where the two FeCl_2_ layers enable the modulation by magnetization. The band structure of intrinsic WSe_2_ shows spin degeneracy, leading to generation of unpolarized photocarrier under light irradiation, namely the amount of spin‐up and spin‐down is the same. In FeCl_2_, the spin‐up (spin‐majority) bands cross the Fermi level, achieving the metallic band structure (Figure S2, Supporting Information). In contrast, the spin‐down (spin‐minority) bands show a direct bandgap beyond 4 eV. The big difference between spin‐up and spin‐down bands makes half‐metal FeCl_2_ a perfect spin filter.

The ability of spin filtering of FeCl_2_ is first evaluated by the spin‐dependent Schottky barrier according to the band structure in WSe_2_/FeCl_2_ heterostructure. As shown by Figure 2b,c, the band structures of intrinsic FeCl_2_ and WSe_2_ (Figure S2, Supporting Information) can still be clearly distinguished in those of the heterostructure, reflecting the absence of interlayer bonding or hybridization at the vdW gap. In spin‐up bands (Figure 2b), due to the metallic bands of FeCl_2_, the Schottky barriers of spin‐up electrons (*Φ* ^*e*^) or holes (*Φ* ^*h*^) are determined by the difference between the Fermi level and conductance bands bottom (CBB) or valance bands top (VBT) of WSe_2_. In contrast, spin‐down bands of FeCl_2_ possess larger bandgap compared with WSe_2_ (Figure 2c), resulting in the determination of *Φ* ^*e*^ and *Φ* ^*h*^ based on the CBB and VBT of spin‐down bands of FeCl_2_. The detailed values are listed in **Table** **1**. Both electrons and holes show smaller Schottky barrier with spin‐up polarization, indicating the easier transport of spin‐up carriers in the heterostructure. This conclusion is further supported by the calculated tunneling probability according to the effective potential. Figure 2d,e exhibit the effective potential of spin‐up and spin‐down electrons. The barrier height for tunneling is determined by the potential difference, *Φ*_TB_ = *e* (*V*_inter_ − *V*_deter_), where *V*_inter_ and *V*_deter_ represent the highest potential of interlayer vacuum layer and determining side, respectively.^[^ ^34^, ^35^ ^]^ For spin‐up electrons, the WSe_2_ with bandgap acts as the determining side (Figure 2d), while the FeCl_2_ with larger bandgap acts as the determining side for spin‐down electrons (Figure 2e). Correspondingly, *Φ*_TB_ and the barrier width *W*_TB_ for spin‐up and spin‐down electrons are extracted and summarized in Table 1. The tunneling probability is therefore obtained by $P_{\text{TB}}{\text{ = e}}^{\left(-\;\frac{2W_{\text{TB}}}{\hbar }\sqrt{\text{2m}{\text{Φ}}_{\text{TB}}}\right)}$, where *w*_TB_ is the width of tunneling barrier, *m* is the mass of the free electron and *ℏ* is the reduced Planck's constant.^[^ ^36^ ^]^ It can be clearly found that the spin‐up electrons show the tunneling probability of 22.45%, much higher than that of spin‐down electrons, which is 0.0035%. Such a big difference suggests the easier tunneling for spin‐up electrons, which can also be drawn from the spin‐dependent density of states (Note S1, Supporting Information).

**Table 1:** Spin‐dependent Schottky barriers of electrons (*Φ* ^*e*^) and holes (*Φ* ^*h*^), barrier height (*Φ*_TB_), width (*W*_TB_), and probability ($P_{\text{TB}}$) of tunneling in WSe_2_/FeCl_2_ heteorstructure.

	*Φ* ^*e*^ [eV]	*Φ* ^*h*^ [eV]	*Φ*_TB_ [eV]	*W*_TB_ [Å]	*P*_TB_ [%]
Spin‐up	1.59	0.11	2.04	1.02	22.45
Spin‐down	4.01	1.05	14.4	2.64	0.0035

**Figure 3:**
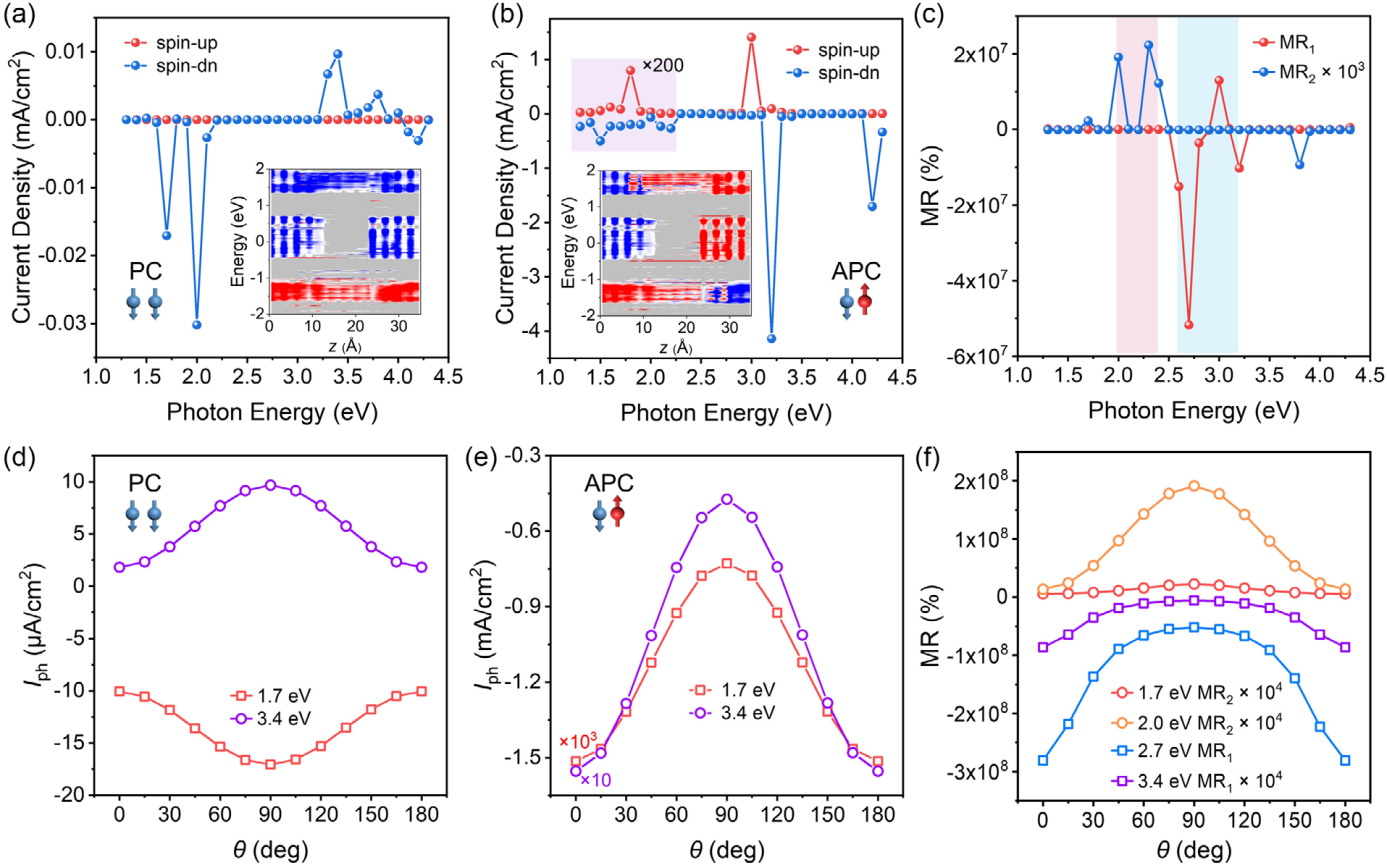
Photocurrent modulated by magnetic configurations. a,b) Current density of spin‐up and spin‐down photocurrent under different photon energies with PC and APC. The polarization angle is 90°. The insets show the schematic of magnetic configurations and the spin‐dependent local projection density of states, where spin‐up and spin‐down are represented by red and blue, respectively. c) Defined values of MR_1_ and MR_2_ under different photon energies. d,e) Polarization angle *θ*‐dependent photocurrent (*I*_ph_) under the photon energies of 1.7 eV and 3.4 eV with PC and APC. f) Polarization angle *θ*‐dependent MR under representative photon energies.

The excellent spin filtering ability of FeCl_2_ produces the reconfigurability in the device. The generation of spin‐dependent photocurrent of PC and APC is displayed in **Figure** **3**a,b respectively, which is under the irradiation of linearly polarized light with the polarization angle of 90° and zero bias. In Figure 3a, the magnetizations of two FeCl_2_ are aligned downward in parallel, making the spin‐down as the spin‐majority. It can be clearly observed that the spin‐up photocurrent is nearly zero all over the photon energy from 1.3 to 4.3 eV, corresponding to the wavelength from 954 to 288 nm. Only the spin‐down photocurrent is generated, showing two most significant ranges of photon energy from 1.7 to 2.1 eV and from 3.3 to 4.2 eV, which can be supported by the projected local density of states (PLDOS) shown in the inset of Figure 3a. The absence of spin‐up electrons around Fermi level indicates the prohibited passing, exhibiting clear comparison with the spin‐down electrons. Besides the PLDOS, the fully spin‐polarized photocurrent can also be contributed by the spin dependent scattering. When the spin polarization of electrons is parallel to the magnetization orientation, the scattering probability is low. When the relationship is antiparallel, the condition is opposite, due to the effect of exchange coupling. In PC case, the spin‐up electrons are strongly scattering, resulting in a pure spin‐down photocurrent. The generation of photocurrent from below 2.1 eV, around the intrinsic direct band gap of WSe_2_ (≈1.5 eV), is mainly attributed from the transition between the bottom of conduction bands and the top of valance bands. Meanwhile, the spin‐majority bands of FeCl_2_ are near the top of valance bands of WSe_2_ in the heterostructure (Figure 2b). The transition from the spin‐majority bands of FeCl_2_ to the conduction bands of WSe_2_ can also contribute to the excitation of photocurrent in this range of photon energy. The smaller energy between these bands even enables the excitation by the energy lower than the intrinsic direct band gap of WSe_2_, extending the applicable wavelength to the near‐infrared. The excitation above 3 eV is mainly caused by the transition at other *k* points, induced by the higher photon energy. The corresponding spin polarization is also strictly confined by the strong spin filtering effect of FeCl_2_. The only generation of spin‐down photocurrent can also be observed with the polarization angle of 0° (Figure S5, Supporting Information), demonstrating the robustness of the spin filtering effect. The case without the consideration of magnetism is also revealed to further support the origin of these excitations. Both the photon energy and current density are similar with the condition of PC (Figure S6, Supporting Information).

While for the case of APC, the excitation around 2 eV with APC shows similar current density compared with PC. Differently, both spin‐up and spin‐down photocurrent can be excited, flowing in opposite directions, which is aligned with the antiparallel magnetizations of FeCl_2_ at two leads. The PLDOS in the inset of Figure 3b with opposite spin polarization at two leads further demonstrates the generation of spin‐polarized photocurrent, which is also consistent with the spin‐dependent scattering effect. The simultaneous switching of the magnetization in FeCl_2_ at two leads can reverse PLDOS and directions of spin‐polarized photocurrent due to the spin filtering effect (Figure S7, Supporting Information). At higher photon energy around 3 eV, the much stronger photocurrent is generated with APC, which is two orders higher than that at lower energy or the condition with PC. Such big difference on the photocurrent indicates the different origin. According to the band alignment in FeCl_2_/WSe_2_ heterostructure, besides the transition inside WSe_2_, the transition from the valance bands of spin‐minority in FeCl_2_ to the conductance bands of WSe_2_ can be excited by the photon energy above 3 eV (Figure 2c). With the antiparallel aligned magnetizations of two FeCl_2_, the generated spin‐minority photocurrent can flow toward the opposite lead. Therefore, the 100% spin‐polarized photocurrent generated at both low and high photon energy flows in the same direction (Figure 3b). In contrast, the parallel aligned magnetizations in PC forbid the transport of spin‐minority photocurrent, creating the big difference on photocurrent between PC and APC at high photon energy. Despite of the strongest excitation of photocurrent around 2 and 3 eV, the total photocurrent is distinguishable in the whole range of photon energy from the ultraviolet to the near‐infrared with tunable amplitude and direction by the magnetic configuration (Figure S8, Supporting Information), reflecting the broadband application of FeCl_2_/WSe_2_/FeCl_2_ structure.

The significant difference of photocurrent defined by the magnetic configuration naturally results in large magnetoresistance (MR), which is generally used to reflect the ability of spin filtering in spin valves. Two kinds of MR are defined here, where MR_1_ = (*I*_APC_ − *I*_PC_)/*I*_PC_ and MR_2_ = (*I*_PC_ − *I*_APC_)/*I*_APC_. As illustrated in Figure 3c, MR_2_ exceeds 10^4^% under the photon energy of intrinsic excitation in WSe_2_, and MR_1_ reaches over 10^7^% under the photon energy of excitation in the heterostructure. The colossal MR reflects the excellent spin filtering effect in our devices, which brings about the strong reconfigurability defined by the magnetic configuration.

In addition to the amplitude of photocurrent, the magnetic configuration also modulates the polarization sensitivity under different photon energies. With PC and APC, dependence of photocurrent (*I*_ph_) on the polarization angle (*θ*) under selective photon energies are shown in Figure 3d,e, respectively. In PC, *I*_ph_ shows a cosine dependence on *θ* and reaches maximum at *θ* = 90° under the photon energies of both 1.7 and 3.4 eV, representing the two ranges of photon energy for excitation. The different current direction between these two photon energies results in the opposite *I*_ph_. Such *θ*‐dependence is consistent with the situation without the introduction of magnetization due to the similar symmetry of the system. In APC, the dependence of *I*_ph_ on *θ* shows a different tendency compared with PC. As shown in Figure 3e, under both 1.7 eV and 3.4 eV, *I*_ph_ also shows a cosine dependence on *θ*, which is maximized at *θ* = 0°. Such difference is resulted by the different magnetic symmetry in APC and PC. In APC, the magnetic mirror symmetry is conserved in *y‐z* plane, which is broken in PC. Therefore, based on the modulation of symmetry in the system by magnetic configuration, the polarization dependence can be effectively modulated. Additionally, through fitting, we can obtain the forms of photocurrent curves under both PC and APC, that is

(1) $$I_{1\text{.7 eV (PC)}}\text{ = }-13\text{.56 +3.50 cos (2}\text{θ}\text{)}$$

(2) $$I_{3\text{.4}\text{eV (PC)}}\text{ = 5.75 }-\text{ 3.93 cos (2}\text{θ}\text{)}$$

(3) $$I_{1\text{.7 eV(APC)}}\text{ = }-1\text{.12 }-\text{ 0.39 cos (2}\text{θ}\text{)}$$

(4) $$I_{3\text{.4 eV(APC)}}\text{ = }-1\text{.01 }-\text{ 0.54 cos (2}\text{θ}\text{)}$$

It indicates that the photocurrent's response to the polarization angle of linearly polarized light follows a cosine relationship, which is consistent with the linear photogalvanic effect (LPGE) theory.

Correspondingly, the value of MR also shows clear dependence on *θ*, which is summarized in Figure 3f. With lower photon energy around 2 eV, positive MR_2_ is achieved, maximum of which approaches 2 × 10^4^% at *θ* = 90°. In contrast, a much larger negative MR_1_ appears with higher photon energy around 3 eV. Due to the opposite *θ*‐dependence *I*_ph_ between PC and APC, MR_1_ reaches its maximum at *θ* = 0°, which is beyond 10^8^%.

### 2.3 ONNs for Multidimensional Recognition

**Figure 4:**
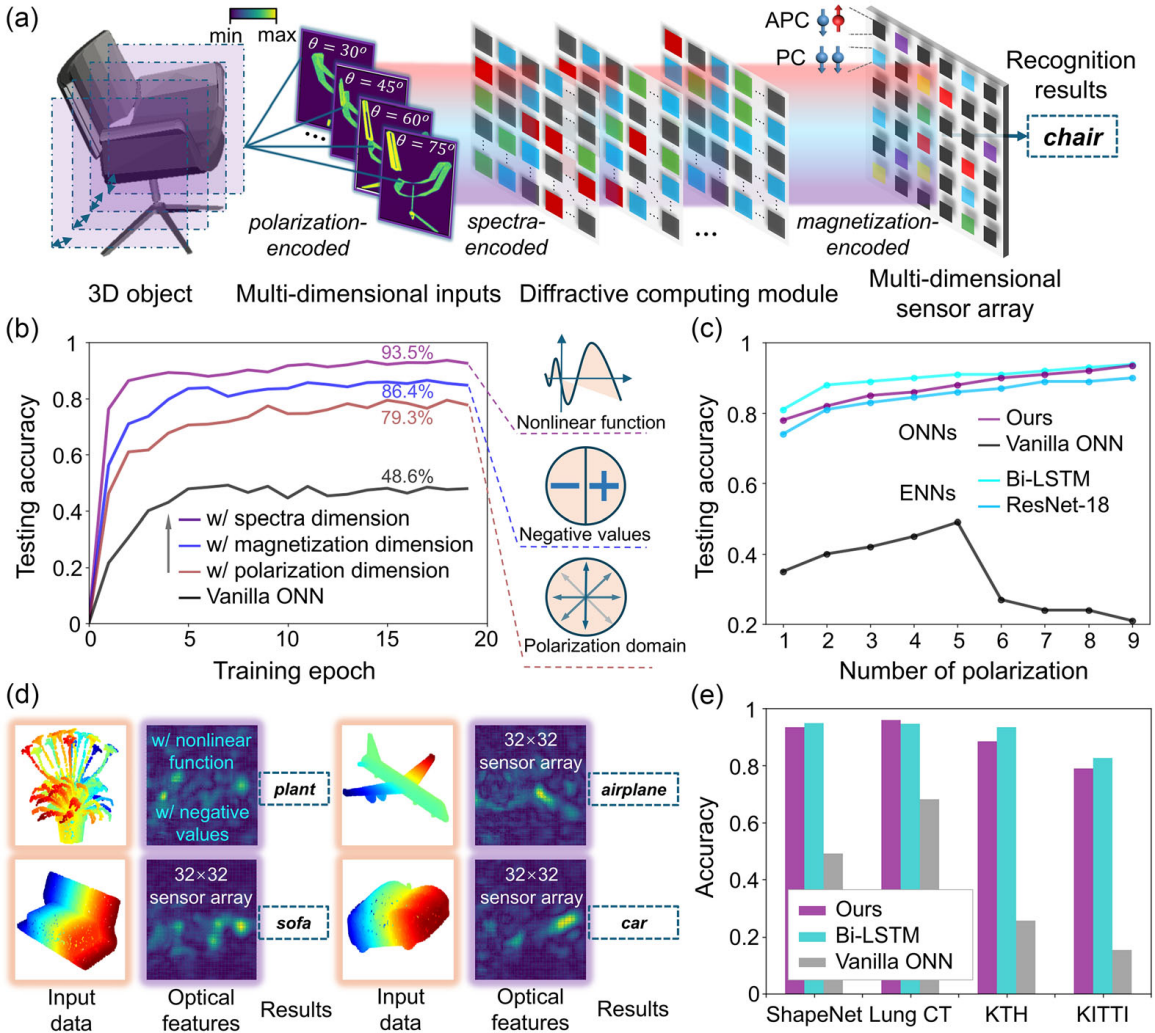
Optical neural network with multidimensional in‐sensor computing. a) Inference process of a 3D object classification task with its projection‐diffraction‐prediction workflow. The multidimensional data is encoded onto light with polarization, spectra, and magnetization information, propagated with free‐space diffractive modulators, and finally read out by a sensor array with multimode in‐sensor computing. b,c) Numerical evaluations of the proposed ONN architecture. Ablation study on different information dimensions, and number of polarizations among optical and electronic methods. d) Visualization of sampled optical features during 3D task propagation. e) Accuracy comparisons among different datasets.

For evaluation of the proposed magneto‐optoelectronic devices, we construct a 32 × 32 array supporting in‐sensor computing for ONNs. **Figure** **4**a presents the overall workflow of the designed free‐space optical architecture, the multichannel representations of inputs are encoded onto light with various dimensions. Multispectral laser and spatial light modulator are employed to produce and guide the diffraction‐based light propagation. The entire system is designed to operate across a wavelength range from ultraviolet to near‐infrared, providing a broad spectrum for selection. The diffractive computing module is a stack of several layers, where each transmits optical features as the inputs to the next (detailed in Experimental Section). Empowered by the multidimensional computational characteristics with polarization, magnetization, and spectrum, the proposed optical architecture enables high‐dimensional intelligent tasks that conventional ONNs cannot achieve.

The numerical evaluations are conducted on the 3D dataset ShapeNet, which contains 55 common object categories with over 50, 000 unique 3D models. We primarily select five classes as a subset for functional validation, each 3D model for inputs is cropped into *l* slice and all resized to 800 × 800 resolution, where *l* is primarily set as 9 in experiments. Multichannel inputs are encoded with positive and negative values, nine different polarization angles in the range of 30°–150°. Note that we perform the ablation study with various configuration of polarization and then determine the best setting to be 15°. The convergence plots with different computational dimensions activated by device features are compared in Figure 4b. We observe that each data dimension of nonlinear function, negative values, and polarization domain significantly enhances network performance, together supporting an advanced task that excludes vanilla ONN with constrained computational capability. The ablation study on the number of polarization angles is reported in Figure 4c, we discover that applying more channels improves the accuracy but approaches equilibration when >5, which represents that parallelism matters a lot in a manageable range.

Figure 4d visualizes sampled optical amplitudes at the sensor plane during network inference. After further propagating with the in‐sensor computing unit, the classification output is detected by spatial patterns. More comparisons among the proposed architecture, vanilla ONN,^[^ ^37^ ^]^ and electronic methods^[^ ^38^, ^39^ ^]^ on various datasets^[^ ^40^, ^41^, ^42^ ^]^ are simulated in Figure 4e (see detailed dataset preparation and training strategy in Note S2, Supporting Information). Versatile challenging applications on 3D object classification, spatial‐temporal recognition, and object detection have demonstrated the general computing capability of proposed multidimensional magneto‐optoelectronic devices (see confusion matrices in Figure S12, Supporting Information), providing a parallel and scalable architecture to practically tackle large‐scale intelligent tasks optically.

## 3 Conclusion

In conclusion, to achieve high‐performance 3D recognition in machine vision systems, the 2D magneto‐optoelectronic device of FeCl_2_/2H‐WSe_2_/FeCl_2_ is proposed, showing polarity sensitivity, low power consumption, wide wavelength detection and significant reconfigurability. The symmetry breaking induced PGE enables the modulation of photocurrent by the light polarization with the wavelength from ultraviolet to near‐infrared at zero bias voltage. Both the amplitude, direction and polarization angle‐dependence of photocurrent are significantly modulated by the magnetic configuration, benefiting from the perfect spin filtering effect of half‐metal FeCl_2_ and the band alignment in the heterostructure. The magneto‐optoelectronic device largely enhances the performance of ONN in 3D object recognition and classification, showing similar capability compared to electronic methods. Our work reveals the potential of high‐dimensional ONN in real‐life applications by taking advantage of magneto‐optoelectronics.

## 4 Experimental Section

4.1

4.1.1

##### Calculations of photocurrent

Our calculations are performed using the first‐principles calculation combined with nonequilibrium Green's function method implemented in the Atomistix ToolKit (ATK) package. The photocurrent is calculated based on a linear response theory.^[^ ^43^ ^]^ In particular, for linearly polarized light the photocurrent injecting into the lead can be written as

(5) $$I_{\text{ph}}=\frac{ie}{h}\int \{{\text{cos}}^{2}\theta \;\text{Tr}\{\Gamma_{L}[G_{1,\text{ph}}^{<}+f_{L}(G_{1,\text{ph}}^{>}-G_{1,\text{ph}}^{<})]\}+\;{\text{sin}}^{2}\theta \;\text{Tr}\{\Gamma_{L}[G_{2,\text{ph}}^{<}+f_{L}(G_{2,\text{ph}}^{>}-G_{2,\text{ph}}^{<})]\}+\frac{\text{sin }\left(2\theta \right)}{2}\text{Tr}\{\Gamma_{L}[G_{3,\text{ph}}^{<}+f_{L}(G_{3,\text{ph}}^{>}-G_{3,\text{ph}}^{<})]\}\}dE$$

where $G_{1,2,3,\text{ph}}^{>/<}$ are the greater/lesser Green's functions with electron–photon interaction, which are determined by the symmetry, photon frequency *w*, and polarization vector *e* as well.^[^ ^44^ ^]^ For linearly polarized light, the polarization vector $e=\cos\theta \;e_{1}+\sin\theta \;e_{2}$, where θ is the angle formed by the polarization direction with respect to the vector $e_{1}$.

The response of photocurrent to polarization angle can be phenomenologically explained according to the standard phenomenological theory. The LPGE produced when linearly polarized light irradiates materials with *C*_s_ symmetry^[^ ^36^ ^]^ can be described by the following formula^[^ ^43^ ^]^

(6) $$j_{x}=E_{0}^{2}(\chi_{+}+\chi_{-}\cos\text{ (}2\theta ))\;$$

(7) $$j_{y}=E_{0}^{2}\chi_{zzx}\text{sin(}2\theta )$$

here $j_{x}$ and $j_{y}$ are the optical current parallel and perpendicular to the specular reflection direction respectively, $E_{0}^{2}$ are the electric field amplitude of light, $\chi_{\text{zz}x}$, $\chi_{+}$, and $\chi_{-}$ are the tensor dependent on the photon frequency, essentially the PGE is the second order response to the electric field.

##### First principles calculations

The generalized gradient approximation with the Perdew–Burke–Ernzerhof form is adopted for the electronic exchange correlation functional. The kinetic energy cutoff for the plane‐wave basis set is 80 Hartree, and the convergence threshold for the force tolerance is less than 0.01 eV Å^−1^. In the calculations of transport properties, the Monkhorst‐Pack k‐meshes of devices are 1 × 8 × 161, the integration grid of the current calculation is 1 × 14, and the convergence criteria of the energy forces is set to10^−5 ^eV. All the parameters are tested to be sufficient to obtain accurate results.

##### Free‐Space ONN Architecture Design and Simulation

The proposed architecture for multidimensional optical learning utilizes sparse optical diffractive modules. In our simulations, each free‐space photonic computing module is comprised of multiple 800 × 800 sparse optical diffractive layers in the Fourier plane of a 4*f* optical system under coherent light. In practice, the number of layers is set corresponding to task complexity (five‐layer for 3D object classification and eight‐layer for spatial‐temporal recognition tasks). The photonic neuron connections are guided and modulated by combination of beam splitters (BS), mirrors (M), lenses (L), and optical filters. Phase modulation and light diffraction are utilized to extract and process optical features, and an optical intensity sensor is employed at the output plane to capture the final results. Multichannel inputs can be encoded onto multidimensional representations, projected to a shared domain, and processed through light diffraction. Recognition results are obtained through an electronic read out layer of 1024 × 6 fully‐connected computation.

The proposed ONN is simulated to work in the polarization angles ranging from 30° to 150°, and wavelengths ranging from ultraviolet to near‐infrared. The N.A. and focal length of the first and second 2f systems are set to be the same: $N\text{.A}{\text{.}}_{1}\text{ = N}\text{.A}{\text{.}}_{2}\text{ = 0}\text{.112}$, $f_{1}\text{ = }f_{2}\text{=10 mm}$. Considering the physical fabrication and N.A. matching, the photonic neuron size of the network is set to 2 μm to achieve sufficient sampling rate for the modulation layer. Specifically, a sparse optical diffractive layer is constructed using four fundamental units: free‐space propagation, phase modulator, sensor, and remapping. Free‐space propagation unit is formulated by the angular spectrum method with *x*‐*y* plane size of 800 × 800, where zero paddings are further adopted to ensure the boundary condition of free‐space propagation. Phase modulator unit applies phase shifts to the input optical field, where number of the trainable photonic neurons is also set to 800 × 800. A *sigmoid* function is employed to constrain the phase modulation range to *0‐2π*. Sensor unit transfers the complex optical information of amplitude and phase to intensity. The intensity‐to‐pixel value mapping is linear due to the gamma correction set as 1. Remapping unit converts the normalized intensity back to complex optical field as input for the following layers. Here we adopt the remapping method from L^2^ONN.^[^ ^8^ ^]^

During training, the loss function is defined as

(8) $$L=L_{\text{CEN}}\left(P\;,G\right)+\alpha \sum_{i=1}^{n}({\left|\left|I_{k}^{i}\right|\right|}^{2}+\;{\left|\left|M_{k}\right|\right|}^{2})$$

where $L_{\text{CEN}}$ represents the cross‐entropy loss, P and G are the network precision and ground truth, and *α* denotes the normalization coefficient, respectively. Moreover, a cosine nonlinear function is implemented onto the proposed ONN, matching the specific response curves in Figure 3.

We apply the intensity mask measured by sensor unit as photonic neuron activation map. For all selected polarization angles, the optical filter initially learns a dense activation map, which is further pruned to a sparse one utilizing an intensity threshold t

(9) $${\text{m}}^{i}[m^{i}<\text{t}]=0$$

where ${\text{m}}^{i}$ denotes the trained map on *i*‐th angles. Only the photonic neurons of intensity beyond threshold will remain activated and keep fixed in the following evolution of learning

(10) $$\Delta O[m^{i}\;\wedge \;\vee_{n=1}^{i‐1}{\text{m}}^{n}]=0$$

where ΔO denotes the gradient matrix of backpropagation on optical weights O, operation ∧ searches the indices of coincident cells between new and former maps, and operation ∨ gradually merges the photonic neurons on activation maps of all polarization angles.

## Supporting Information

Supporting Information is available from the Wiley Online Library or from the author.

## Conflict of Interest

The authors declare no conflict of interest.

## Supporting information

Supplementary Material

## Data Availability

The data that support the findings of this study are available from the corresponding authors upon reasonable request.
