# Supplementary material for: Reconfigurable Magneto‐Optoelectronic Devices for Multidimensional Optical Neural Network
Source: Small Sci. 2025 Dec 7;6(1):e202500487. doi: 10.1002/smsc.202500487 (PMC12798793; doi:10.1002/smsc.202500487)
Supplement: Supplementary file 1 — Supplementary Material [file SMSC-6-e202500487-s001.pdf]

# Supporting Information

**Reconfigurable Magneto-Optoelectronic Devices for High-Dimensional Optical Neural Network**

*Haiyan He, Yuan Cheng, Wenxuan Zhu*, Jiacheng Sun, Jiaming Sun, Tonglu Wang, Cheng Song, Feng Pan, Junying Zhang*, Yuyan Wang**

**Note S1. Demonstration of spin-filtering effect by the spin-dependent density of states.**

The calculations of spin-dependent differential charge density (DCD) can further reflect the spin-polarized charge transfer in $WSe_2/FeCl_2$ heterostructure. As shown in Figure S3a, the spin-up (spin-majority) charge shows a clear depletion around $FeCl_2$, compared with the accumulation of spin-down (spin-minority) charge, indicating the easier transfer of spin-up carrier from $FeCl_2$ to $WSe_2$. According to the obvious change of DCD around $FeCl_2$, the density of states (DOS) of Fe atoms in $FeCl_2$ before and after the construction of heterostructure are calculated for comparison, exhibited in Figure S4a and b, respectively. It is clearly found that the spin-up DOS of Fe is reduced after the construction of heterostructure with $WSe_2$, supporting the transfer preference of spin-up carrier in the heterostructure.

**Note S2. Dataset preparation and training strategy**

For the evaluation on free-space multi-dimensional ONN, to prepare the KITTI dataset,[1] the implementation applies an object subset with 7,500 images for training and 2,500 images for validation (see learning strategy and training method in Figure S10). The original 1225 × 375 images were divided into 2 patches, each with 612 × 187 pixels. The patches were sampled with boundary padding to match the network input size of 800 × 800. We randomly selected 15,000 patches for training, 5,000 patches for validation, and 3,000 patches for testing. All images were represented with RGBD (red, green, blue and depth) channels and encoded onto different polarization angles. In our ONN implementation we selected four representative photon energies: 1.7 eV, 2.0 eV, 2.7 eV, and 3.4 eV (shown in Figs. 3d–f), which span the ultraviolet–visible–near-infrared range where the device exhibits clear tunable magneto-photoresponse. These four wavelengths are used as independent spectral channels, enabling multi-spectral computation analogous to multi-wavelength optical encoding. Each spectral channel contributes a distinct nonlinear activation pattern, effectively broadening the computational bandwidth of the optical layer.

For 3D classification tasks such as ShapeNet, we primarily select 5 classes as a subset for functional validation of the proposed multi-dimensional ONN architecture, each 3D model for inputs is cropped into $l$ slices and all resized to 800 × 800 resolution, where $l$ is primarily set as 9 in experiments. Multi-channel inputs are encoded with 9 different polarization angles in range of 30°-150°, separated by 15°. Similar configurations are applied for Lung CT[2] (Figure S10) and KTH datasets (Figure S11).[3]

The simulation network model is implemented with PyTorch V1.11 running on a single NVIDIA RTX4090 graphic card. Network parameters are optimized using the Adam optimizer.[4] All benchmarks including vanilla ONN,[5] ResNet-18,[6] and Bi-LSTM[7] for comparison are made under the same hardware and software environments.

**References**

[1] A. Geiger, P. Lenz, C. Stiller, R. Urtasun, *Int. J. Rob. Res.* **2013**, *32*, 1231.

[2] M. Ghaderzadeh, F. Asadi, R. Jafari, D. Bashash, H. Abolghasemi, M. Aria, *J. Med. Internet Res.* **2021**, *23*, e27468.

[3] C. Schüldt, I. Laptev, B. Caputo, in *Proc. 17th Int. Conf. Pattern Recognit.*, **2004**, 32.

[4] D. P. Kingma, J. Ba, **2014**, *arXiv preprint arXiv:1412.6980.*

[5] X. Lin, Y. Rivenson, N. T. Yardimci, M. Veli, Y. Luo, M. Jarrahi, A. Ozcan, *Science* **2018**, *361*, 1004.

[6] K. He, X. Zhang, S. Ren, J. Sun, *Proc. IEEE Comput. Soc. Conf. Comput. Vis. Pattern Recognit.*, **2016**, 770.

[7] Sima Siami-Namini, Neda Tavakoli, Akbar Siami Namin, *IEEE Int. Conf. Big Data*, **2019**, 3285.

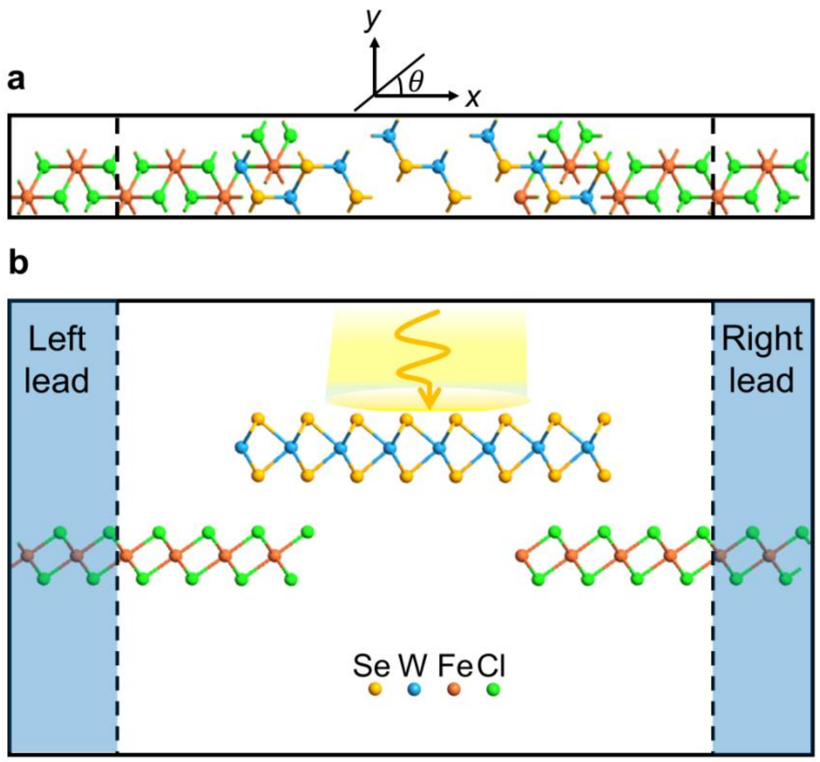


**Figure S1.** a,b) Top and side view of $FeCl_2/WSe_2/FeCl_2$ device.

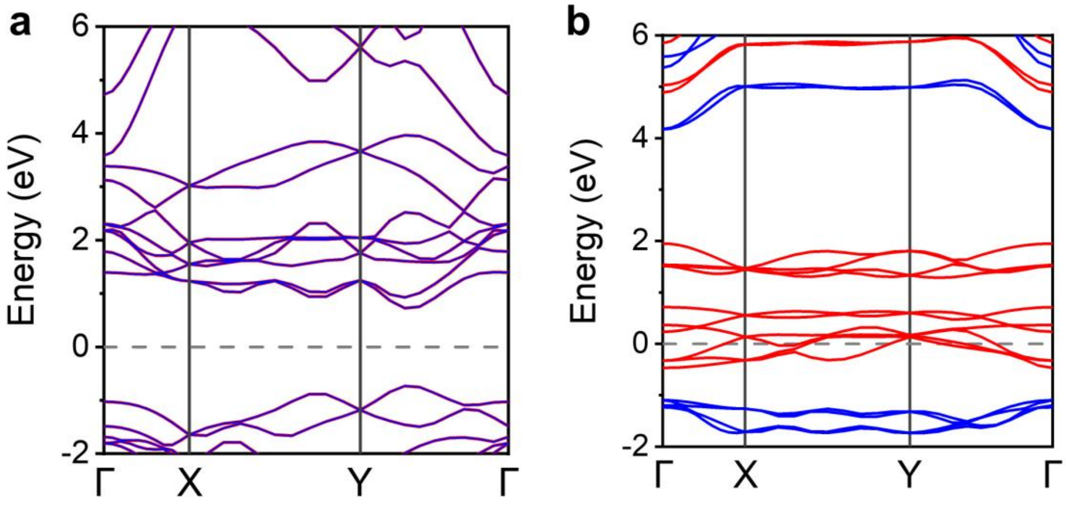


**Figure S2.** Spin-dependent band structures of intrinsic a) $WSe_2$ and b) $FeCl_2$. The spin-up and spin-down bands are shown in red and blue. The Fermi level is set as zero, shown by the dashed line.

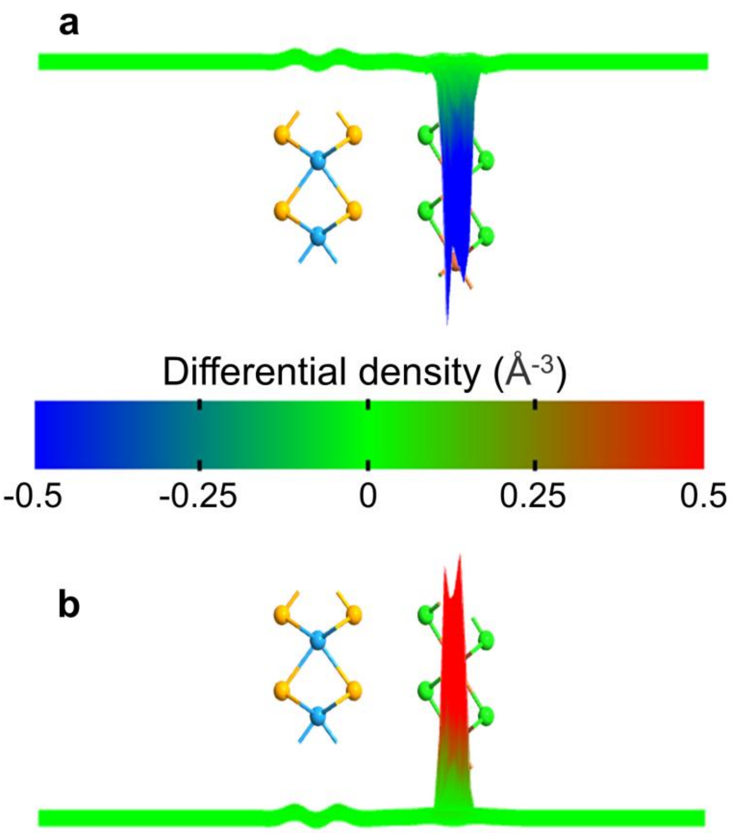


**Figure S3.** Differential charge density of a) spin-up and b) spin-down charge in $WSe_2/FeCl_2$ heterostructure. The red and blue contours represent the charge accumulation and depletion, respectively.

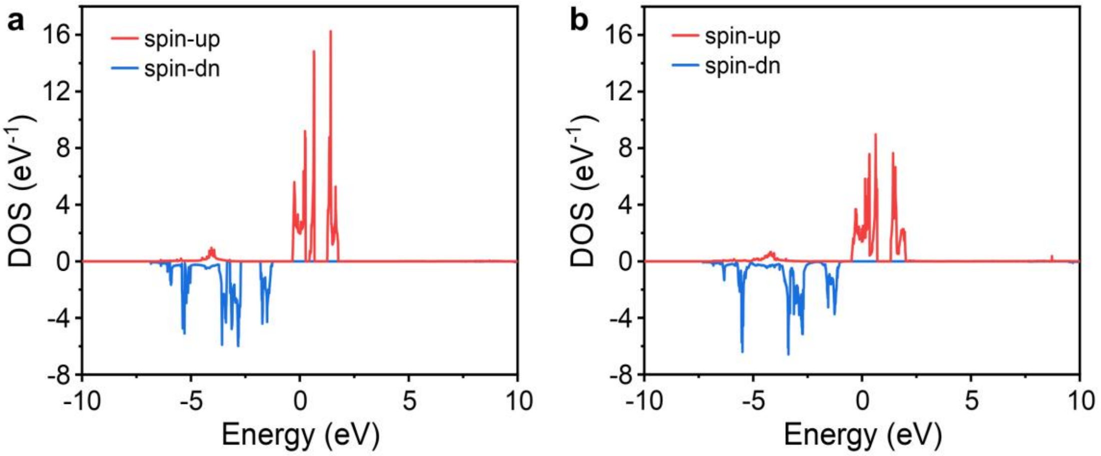


**Figure S4.** Spin-dependent density of states (DOS) of Fe atoms in a) the primitive cell of $FeCl_2$ and b) $WSe_2/FeCl_2$ heterostructure.

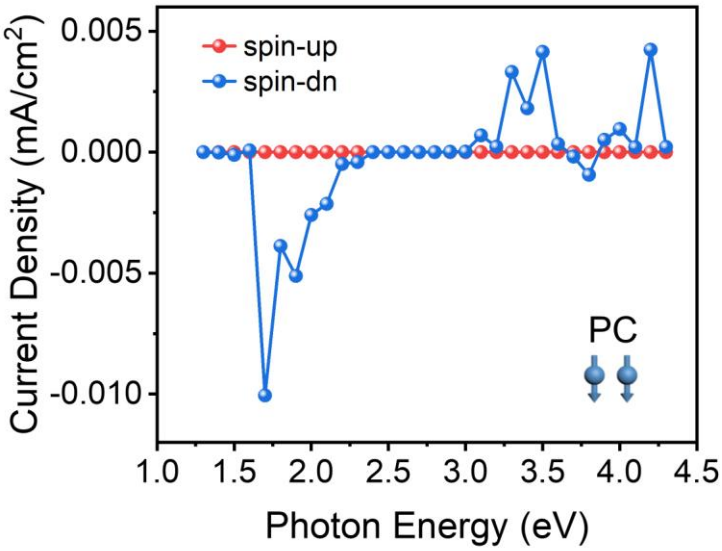


**Figure S5.** Current density of spin-up and spin-down photocurrent under different photon energies with the polarization angle of 0° and PC. The inset shows the schematic of the magnetic configuration.

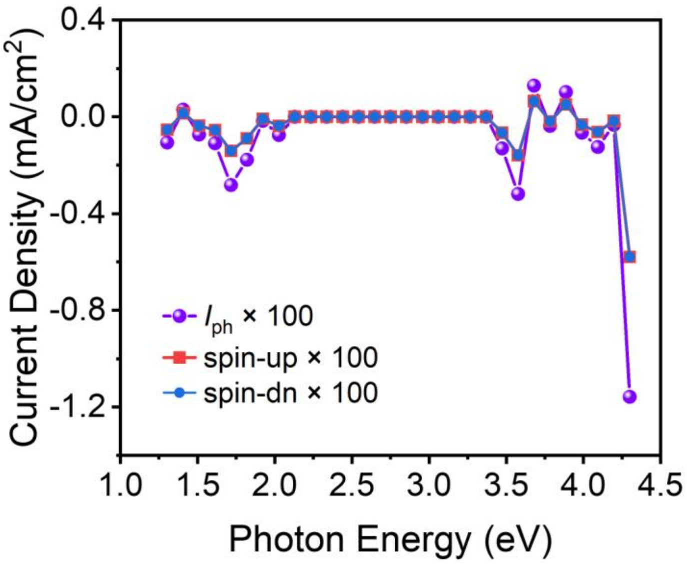


**Figure S6.** Calculated current density of total photocurrent ($I_{ph}$) and spin-dependent $I_{ph}$ in $FeCl_2/WSe_2/FeCl_2$ under different photon energies without the consideration of magnetism.

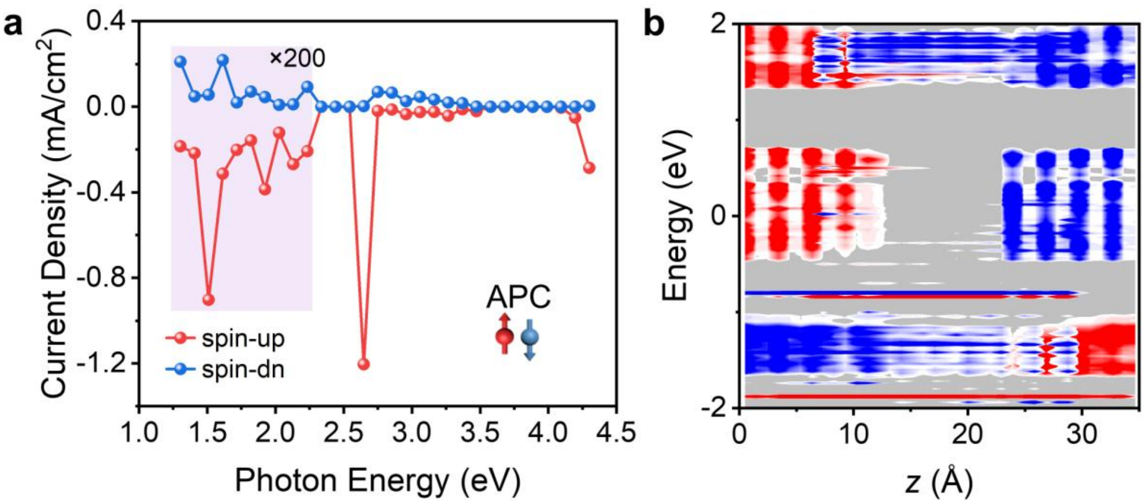


**Figure S7.** a) Current density of spin-up and spin-down photocurrent under different photon energies with APC. The magnetizations at two leads are switched compared with Figure 3b in the main text. The inset shows the schematic of magnetic configuration. b) Spin-dependent local projection density of states with APC and switched magnetizations. The spin-up and spin-down are represented by red and blue, respectively.

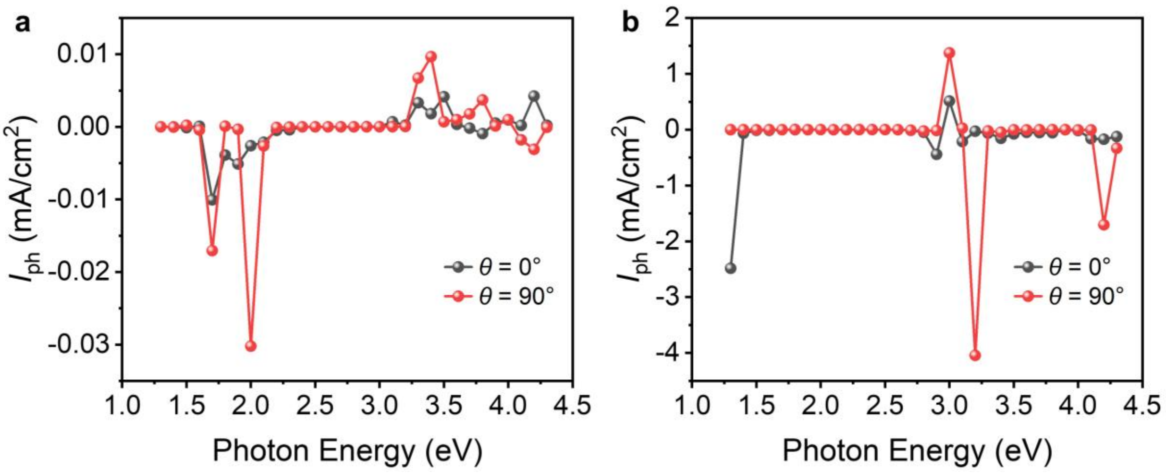


**Figure S8.** Total photocurrent in a) PC and b) APC with the polarization angle ($\theta$) of 0° and 90°.

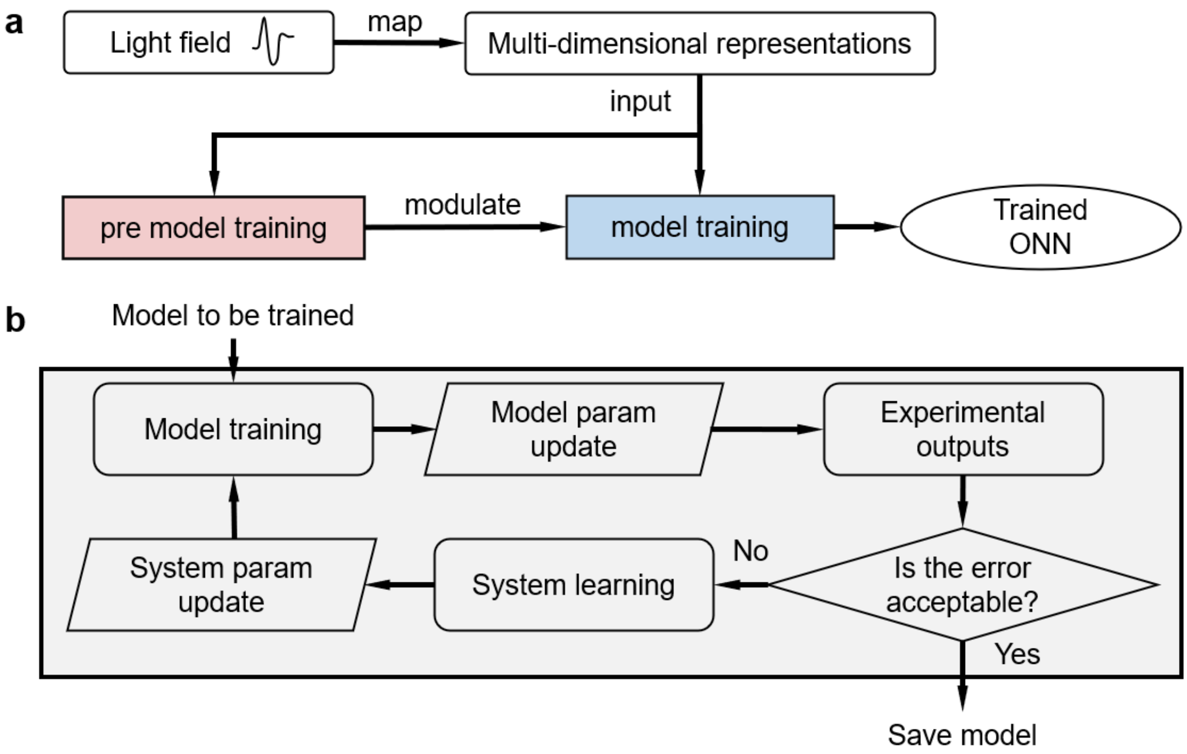


**Figure S9.** Flowchart of a) the multi-dimensional ONN learning strategy and b) training method. The network is trained on an electronic computer with configured system calibration. The proposed adaptive training approach can overcome model deviation and restore inference accuracy by iteratively fine-tuning the network parameters layer by layer. The process of system learning and network training is iterated until the error decreases to an acceptable level.

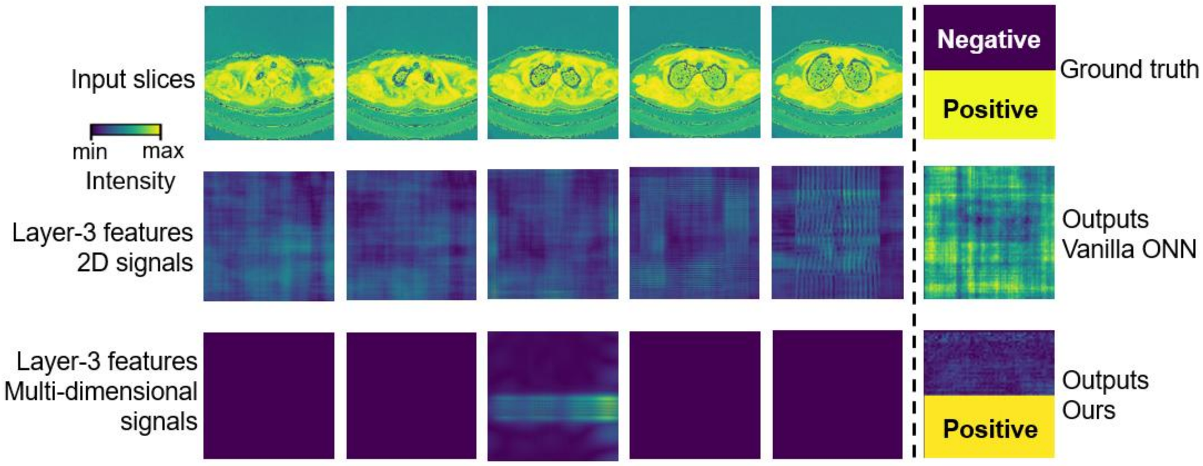


**Figure S10.** Multi-dimensional ONN inference on lung CT diagnosis. Samples of lung CT inputs and their corresponding features in the Fourier space extracted by last layer. The final outputs are detected by sensors and classified with 2 patterns of Negative or Positive. The network version with only 2D signals generates a chaotic map while the one with proposed multi-dimensional signals successfully delighted the desired areas.

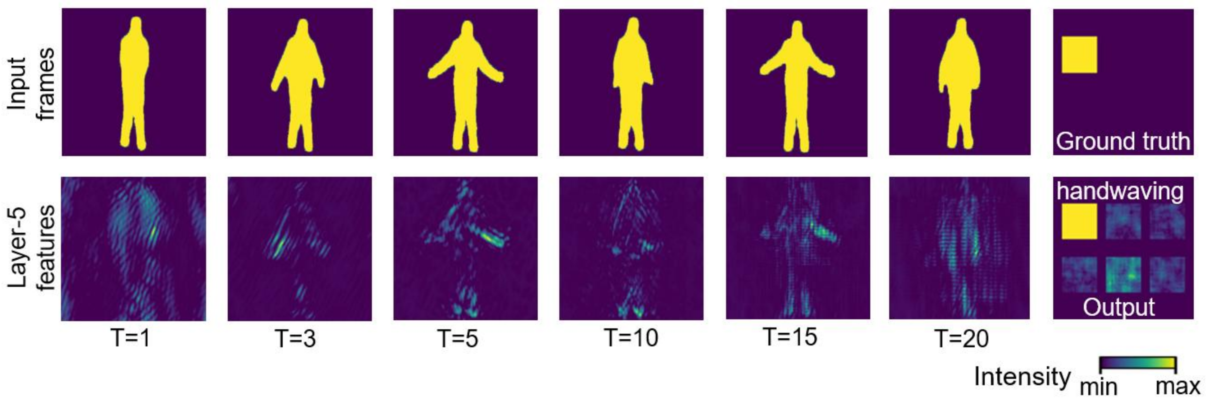


**Figure S11.** Multi-dimensional ONN inference on spatial-temporal tasks. Samples of input frames from KTH dataset and their corresponding features from the last network layer. The proposed ONN adaptively extracts sparse features during inference and recognizes the activity at the sensor plane.

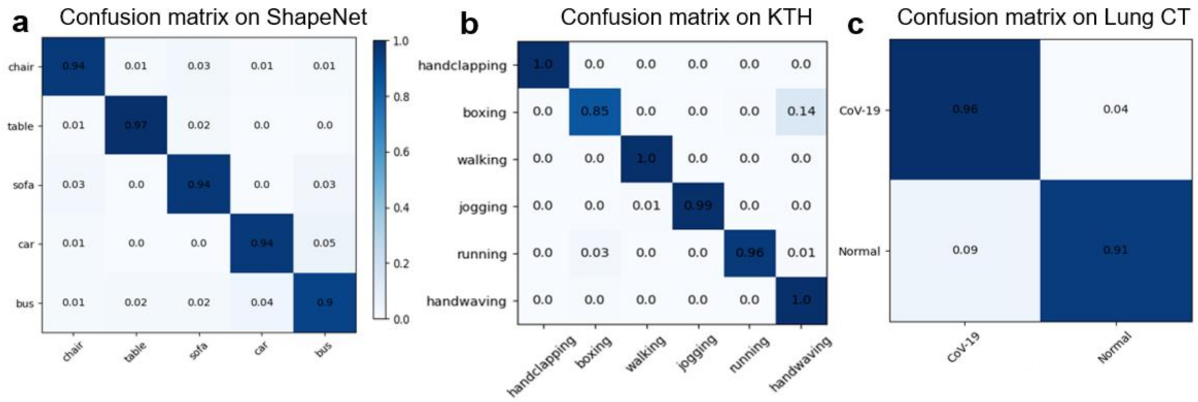


**Figure S12.** Confusion matrices of multi-dimensional ONN on 3 representative complex classification datasets, including a) ShapeNet, b) KTH and c) Lung CT.
